# Application of Metabolomics to Identify Hepatic Biomarkers of *Foie Gras* Qualities in Duck

**DOI:** 10.3389/fphys.2021.694809

**Published:** 2021-07-07

**Authors:** Zohre Mozduri, Bara Lo, Nathalie Marty-Gasset, Ali Akbar Masoudi, Julien Arroyo, Mireille Morisson, Cécile Canlet, Agnès Bonnet, Cécile M. D. Bonnefont

**Affiliations:** ^1^Department of Animal Science, Faculty of Agriculture, Tarbiat Modares University, Tehran, Iran; ^2^GenPhySE, Université de Toulouse, INRAE, ENVT, Castanet Tolosan, France; ^3^ASSELDOR, Station d’Expérimentation Appliquée et de Démonstration sur l’oie et le Canard, La Tour de Glane, Coulaures, France; ^4^Toxalim, Université de Toulouse, INRA, ENVT, INP-Purpan, UPS, Toulouse, France; ^5^Axiom Platform, MetaToul-Me, National Infrastructure for Metabolomics and Fluxomics, Toulouse, France

**Keywords:** liver, quality, biomarker, metabolomics, *foie gras*

## Abstract

*Foie gras* is a traditional dish in France that contains 50 to 60% of lipids. The high-fat content of the liver improves the organoleptic qualities of *foie gras* and reduces its technological yield at cooking (TY). As the valorization of the liver as *foie gras* products is strongly influenced by the TY, classifying the *foie gras* in their potential technological quality before cooking them is the main challenge for producers. Therefore, the current study aimed to identify hepatic biomarkers of *foie gras* qualities like liver weight (LW) and TY. A group of 120 male mule ducks was reared and overfed for 6–12 days, and their livers were sampled and analyzed by proton nuclear magnetic resonance (^1^H-NMR). Eighteen biomarkers of *foie gras* qualities were identified, nine for LW and TY, five specific to LW, and four specific to TY. All biomarkers were strongly negatively correlated to the liver weights and positively correlated to the technological yield, except for the lactate and the threonine, and also for the creatine that was negatively correlated to *foie gras* technological quality. As a result, in heavy livers, the liver metabolism was oriented through a reduction of carbohydrate and amino acid metabolisms, and the plasma membrane could be damaged, which may explain the low technological yield of these livers. The detected biomarkers have been strongly discussed with the metabolism of the liver in nonalcoholic steatohepatitis.

## Introduction

*Foie gras* is one of the flagship products of French gastronomy. It is the product of hepatic steatosis due to the overfeeding of ducks with an energy-rich feed based on corn. The *foie gras* is the result of the increase in triglyceride storage in the liver. Actually, in the small intestine, the degradation of starch from the corn feed led to an accumulation of carbohydrates absorbed into the hepatic portal vein and carried to the liver. There, the *de novo* lipogenesis process converts carbohydrate precursors into fatty acids. During the overfeeding period, the imbalance between the lipid neosynthesized in the liver and their export in the hepatic vein causes lipids to accumulate as triglycerides in the hepatocytes, conducting to hepatic steatosis. Furthermore, some exported triglycerides go back to the liver and are stored in this organ. The first mechanism consists of exporting the newly formed triglycerides to the peripheral tissues like muscles and abdominal or subcutaneous tissues for storage or energy utilization. This process is mediated by very low-density lipoprotein (VLDL; Goodridge, 1987). Then, a part of these exported triglycerides is returned to the liver *via* high-density lipoprotein (HDL) and stored in the liver (Tavernier et al., 2018).

The mule duck is the main represented species for *foie gras* production because it has the best ability to hepatic steatosis (Baéza et al., 2005). The duration of fasting before slaughtering and the conditions of evisceration also play important roles in the *foie gras* quality (Auvergne et al., 1998). Indeed, studies have shown that if the liver evisceration occurs 20 min after slaughtering, and if the cooling of livers is quick, the livers have a high technological yield (TY; Bouillier-Oudot et al., 2004). The TY of *foie gras* that is the opposite of the melting rate has strong repercussions on both the organoleptic qualities of *foie gras* and on the performances of the industrial production units. Thus the TY has already been widely studied (Theron et al., 2013). In ducks, there is also a positive correlation between the liver weight (LW) and the melting rate at cooking, especially above 600 g (Blum et al., 1990; Marie-Etancelin et al., 2011). Similarly, the lipid level correlates with the melting rate at cooking (Rousselot-Pailley et al., 1992). Zootechnical factors, pre-, post-mortem conditions, and liver characteristics have already been analyzed to control better the liver melting at cooking, but some individual variabilities persist (Theron et al., 2012).

In a recent study, plasmatic biomarkers of *foie gras* quality (LW and TY) were identified by proton nuclear magnetic resonance (^1^H-NMR; Mozduri et al., 2021). The plasmatic biomarkers are of main interest because they can bring information of *foie gras* quality before slaughtering the animals. In a second step, hepatic biomarkers of *foie gras* qualities are detected on the same experimental materials. Hepatic biomarkers of crude livers might be very useful to choose the cooking program to apply to livers like pasteurization, sterilization, or emulsion to optimize the *foie gras* products in the industry. Moreover, metabolomics studies with ^1^H-NMR were already performed on livers at the end of the overfeeding period to distinguish the signaling of livers with high fat loss corresponding to low TY from livers with low-fat loss that corresponded to high TY (Bonnefont et al., 2014). However, the livers weighed around 570 g to 582 g. The strength of the present study was that male mule ducks were overfed for 6 to 12 days which provided a wide variety of liver weights from 300 g to over 900 g.

This study aimed to identify hepatic biomarkers specific to LW and TY by metabolomics approach using ^1^H-NMR by analyzing the duck livers after 6 to 12 days of overfeeding. This study can also provide information to understand nonalcoholic fatty liver disease in humans better.

## Materials and Methods

### Animal Experimental Design and Liver Characteristics

**Figure 1:**
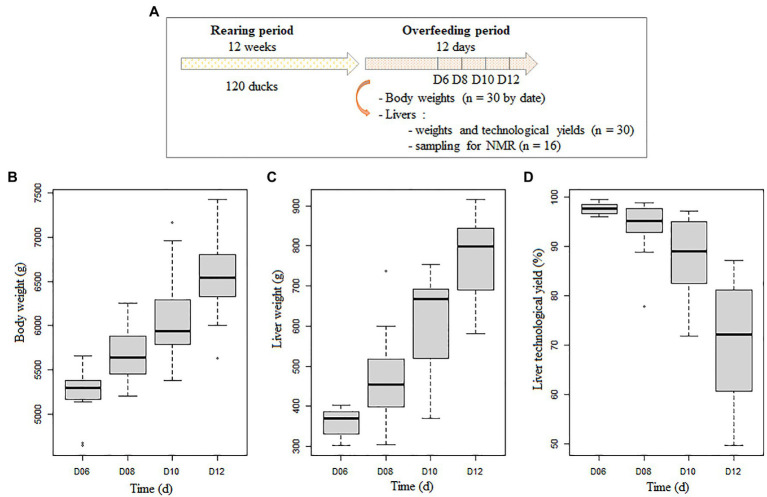
Description of the duck experimental design and the duck characteristics. Experimental design and liver sampling **(A)**, evolution during the overfeeding period of duck body weight before slaughtering **(B)**, of liver weight after eviscerating **(C)** and liver technological yield after cooking (**D**; *n* = 16 or 17 at each time point).

The animal design was clearly described previously (Bonnefont et al., 2019; Mozduri et al., 2021). Briefly, 120 male mule ducks (*Cairina moschata* × *Anas platyrhynchos*) were reared until 12 weeks and overfed twice a day during 6 to 12 days corresponding to 11 to 23 meals. The feed was composed of 97% corn (38% of grain and 62% of flour) supplemented with 3% of a commercial premix diluted into water. The amount of feed increased gradually from 265 to 420 g by meal (Bonnefont et al., 2019). A total of 30 ducks were slaughtered every other day in the second half of the overfeeding period from the 11th meal on day 6 to the 23rd meal on day 12 (Figure 1A). The duck body weight was registered before slaughtering. Then the liver was eviscerated and weighed to obtain LW. Then the livers were cooked as described in Rémignon et al. (2018), and the TY was determined as the ratio between cooked liver weights trimmed of all visible fat and raw liver weights (TY = 100 – % fat loss). At each time point (D6 to D12), 16 ducks were selected among the 30 ducks for liver analyses (*n* = 64 in total). They were chosen to obtain equivalent means and variabilities of LW and TY in the initial and subgroup groups. The selected samples corresponded to the samples used for identifying plasmatic biomarkers (Mozduri et al., 2021). The body weight, LW, and TY of the selected samples are represented in Figures 1B–D.

### Liver Sampling and ^1^H-NMR Analysis

At 20 min *post-mortem*, a sample of 20 g was taken off in the upper part of the main lobe of the liver. All samples were dropped into liquid nitrogen and stored at −80°C for ^1^H-NMR analyses. They were ground into fine powder. Then, their polar metabolites were extracted with a method adapted from Beckonert et al. (2007) from 0.25 g of the crushed liver with methanol and dichloromethane (Beckonert et al., 2007) and carefully described in Mozduri et al. (2021). The upper phases composed of water and methanol with hydrophilic metabolites were collected in new polypropylene tubes and evaporated with a vacuum concentrator (Concentrator Plus, Eppendorf, Hamburg, Germany) and stored at −80°C until ^1^H-NMR analysis. Then all samples were diluted into 650 μl of NMR pH7 phosphate buffer in deuterated water (D_2_O) with sodium trimethylsilyl propionate (17.2 g TMSP for 100 ml). The tubes were vortexed and then centrifuged for 15 min at 5,350 g. Finally, 600 μl were sampled in NMR tubes of 5 mm. The ^1^H-NMR analyses were done by Bruker Avance III HD NMR spectrometer operating at 600 MHz for a proton resonance frequency. The first step consisted of acquiring all spectra. For this purpose, the NOESYPR1D spin-echo pulse sequence was used to attenuate signals from water. The spectra were acquired at 300 K with time domain: 32 k, 16 dummy scans, and 512 scans for all samples. After Fourier transformation, using Topspin (V2.1, Bruker, Biospin, Munich, Germany), they were manually phased, corrected for the baseline, and calibrated with chemical shifts of TMSP at 0 ppm.

### Spectra Preprocessing and Statistical Analysis

The ^1^H-NMR spectra were analyzed by two methods: (i) a bucket method and (ii) a metabolite method, both of which were carefully described previously (Mozduri et al., 2021). (i) Briefly, the traditional bucket method consisted in converting the ^1^H-NMR spectra into a bucket value table with the Workflow4Metabolomics 3.3 online platform (Giacomoni et al., 2015).^1^ After spectra preprocessing (solvent suppression from 5.1 to 4.5 ppm and 3.35 to 3.2, zero-filling, apodization, application of Fourier transform, phasing, baseline correction, and calibration with TSP at 0.0 ppm) and spectra alignment, the spectra were split into buckets with a 0.01 ppm interval from 0.5 to 10 ppm. The raw bucket values were calculated as the integration of the spectrum curves for the corresponding buckets. Then the bucket values were normalized with the integration of the whole spectrum curves as following:

$$\begin{array}{l} \mathrm{Normalized bucket value}=\mathrm{Raw}\;\mathrm{bucket value}/\mathrm{Whole spectrum integration.} \end{array}$$

A table of bucket values was obtained with 64 rows corresponding to the animals and 714 columns corresponding to the buckets identified by their chemical shifts. (ii) Briefly, the metabolite method converted the ^1^H-NMR spectra into metabolite relative concentration tables with the ASICS R package (R package version 4.0.2).^2^ ASICS package performed an automatic approach to identify and quantify metabolites in complex ^1^H-NMR spectra from their unique peak pattern (fingerprint; Tardivel et al., 2017; Lefort et al., 2019). The metabolite database used consisted of the spectra of 176 pure metabolites described in Tardivel et al. (2017). A total of 80 metabolites were identified and quantified in at least one sample. The methanol was removed as it was used to extract the metabolites. Then only 41 metabolites were kept for further analyses as they were present in at least 50% of the samples at one time point. Thus, the final table of metabolite relative concentrations contained 64 rows corresponding to the animals and 41 columns corresponding to the metabolites.

The bucket and metabolite relative concentration tables were analyzed with SIMCA P+ software (version 12, Umetrics, AB, Umea, Sweden) for carrying out the multivariate statistical analysis as previously described (Mozduri et al., 2021). Briefly, the variables were preprocessed with Pareto normalizations. Principal component analysis (PCA) was performed for finding outliers. Then partial least square analyses (PLS) were performed to explain Y variables (LW and TY) by the X variables (bucket or metabolite data). The PLS scatter plots were drawn, but as only one latent variable was created, the PLS scatter plot represented the scores (t1) on the vertical axis vs. sample identification on the horizontal axis. The goodness-of-fit of the models were estimated by the proportion of cumulative explained variance (R^2^) for both the X variables (X = buckets or metabolites) and the Y variable (Y = LW or TY) and by the predictive ability of the model (Q^2^). The root mean square error of estimations (RMSEE) were computed and indicated the fits of the observations to the model. The root mean square error after cross-validations (RMSECv) were also computed. The plot of the Y observed vs. Y predicted values were drawn for each PLS model. The validation of the PLS model was evaluated by comparing the goodness of fit (R^2^Y and Q^2^) of the original model with the goodness of fits of 500 models based on data where the ranks of the Y-observations have been randomly permuted, while the X-matrix (bucket or metabolite) has been kept intact. The permutation plots were drawn. The latent variables associated with interesting axes were analyzed using the variable importance in the projection (VIP) method. The variable (bucket or metabolite) with a VIP superior to 1 was considered as “important.” Then a one-by-one regression with either the LW effect or the TY effect was performed on the whole datasets. The *p*-values were corrected for multiple tests with the Benjamini–Hochberg correction using the R software (version 3.6.1) and named “BH *p*-value.” A variable was “significant” when the BH *p*-value was inferior to 0.05 and “tended to be significant” when the BH *p*-value was between 0.05 and 0.1.

For the buckets with VIP superior to 1, the corresponding metabolites were identified manually by importing the chemical shift lists into the Human Metabolome Database (Wishart et al., 2009).^3^ All carbohydrates identified were D-carbohydrates, and all amino acids were L-amino acids. To simplify the names of the metabolites, the “D-” and the “L-” were removed before the names of the carbohydrates and the amino acids, respectively. To confirm the identification of the metabolites, the ^1^H-NMR peaks of these metabolites were manually checked on the sample spectra with TopSpin software (version 4.0, Bruker BioSpin, Rheinstetten, Germany). For each metabolite, all ^1^H-NMR peaks were listed. For each ^1^H-NMR peak, the VIP values and the BH *p*-values of the corresponding buckets were summarized by the number of buckets with VIP superior to 1 and by the range of BH *p*-values, respectively. The relative concentrations (RC) of a metabolite with the bucket data were estimated with a method adapted from Kostidis et al. (2017) by the following formula (Kostidis et al., 2017):

$$\mathrm{Metabolite}\;{\mathrm{RC}}_{j}=\mathrm{mean}\;\left[\left({\mathrm{intensity Peak}}_{\mathrm{ij}}\right)/\left(H\;{\mathrm{number Peak}}_{\mathrm{ij}}\right)\right]$$

where j represented a specific metabolite, i represented each proton peak of the ^1^H-NMR spectrum of the j metabolite, “intensity Peak_ij_” was computed as the sum of the bucket intensity of the i peak for the j metabolite, and “H number Peak _ij_” was the number of protons corresponding to the i peak for the j metabolite. Then the lists obtained by the bucket method and the metabolite method were compared with Venn diagrams.^4^

All the biomarkers identified by the bucket method and/or the metabolite method were considered as biomarkers. Network analysis based on the correlation of the biomarker RC and the variable (LW or TY) was performed with the functions pls and network of the MixOmics R package (Lê Cao et al., 2009; Rohart et al., 2017; R package version 4.0.2.).^5^

## Results

The overfeeding of male mule ducks from 6 to 12 days enabled to obtain animals with large variability of performances. The body weights of the ducks were between 5 and 7.5 kg, their LW between 302.3 and 914.9 g, and the liver TY between 54.8 and 99.5% (Figures 1B–D). This experimental design enabled to obtain livers with strong variabilities like in the *foie gras* industry, and it was suitable to detect hepatic biomarkers of *foie gras* quality by ^1^H-NMR analysis.

### Identification of Hepatic Biomarkers of the Liver Weight of *Foie Gras*

**Figure 2:**
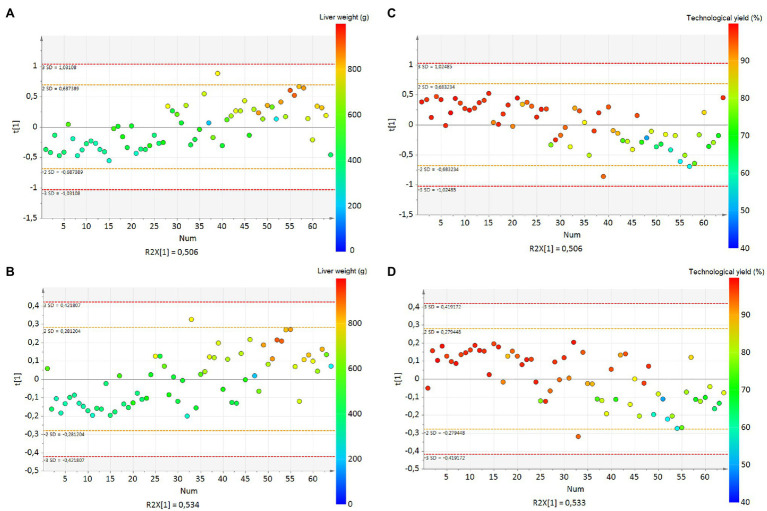
PLS score plots **(A)** for liver weight with the bucket method (R^2^X = 0.506, R^2^Y = 0.657, Q^2^ = 0.633) and **(B)** with the metabolite method (R^2^X = 0.534, R^2^Y = 0.65, Q^2^ = 0.626), **(C)** for liver technological yield with the bucket method (R^2^X = 0.506, R^2^Y = 0.514, Q^2^ = 0.471), **(D)** for liver technological yield with the metabolite method (R^2^X = 0.533, R^2^Y = 0.522, Q^2^ = 0.418). The numbers correspond to the identification of the samples and the colors to the liver weight value. The legend is indicated on the right of the figure. The numbers correspond to the identification of the samples on the X axis and the colors to the liver weight or technological yield values.

**Figure 3:**
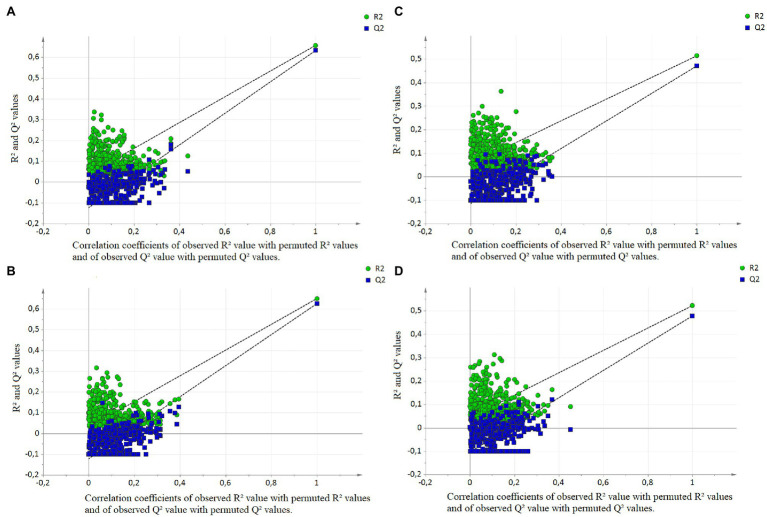
Permutation plots **(A)** for liver weight with the bucket method, **(B)** for liver weight with the metabolite method, **(C)** for liver technological yield with the bucket method, **(D)** for liver technological yield with the metabolite method. 500 permutations were performed. The permutation plot shows, for a selected Y-variable, on the vertical axis the values of R^2^Y and Q^2^ for the original model and of the Y-permuted models. The horizontal axis shows the correlation between the permuted Y-vectors and the original Y-vector for the selected Y. The original Y has a correlation of 1.0 with itself.

**Figure 4:**
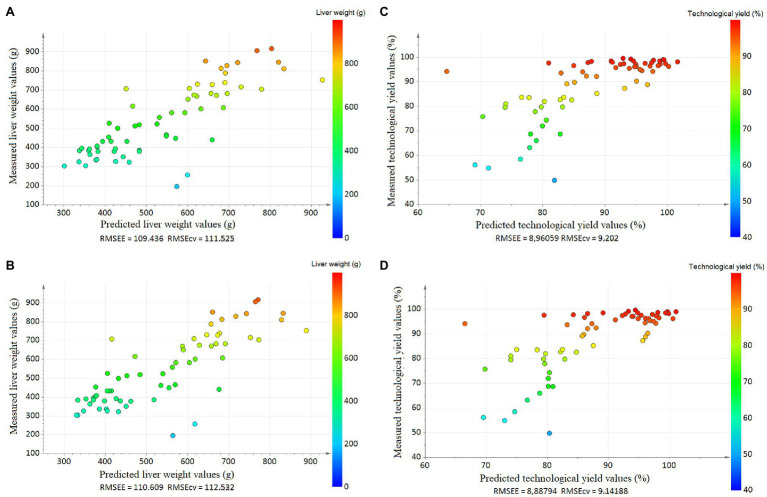
Plots of predicted vs. observed data **(A)** for liver weight with the bucket method, **(B)** for liver weight with the metabolite method, **(C)** for liver technological yield with the bucket method, and **(D)** liver for technological yield with the metabolite method. The Root Mean Square Error of Estimation (RMSEE) and the Root Mean Square Error after cross validation (RMSECv) were indicated.

First, a PCA was implemented to observe the data and to find outlier samples, but no outlier was detected (not shown). The partial least squares (PLS) analysis scatter plot had only one latent variable, and the parameters were cumulative R^2^X = 0.506 and R^2^Y = 0.657. The projection of the samples highlighted an evolution of LW with the first latent variable on the vertical axis (Figure 2A). The prediction of the model was Q^2^ = 0.633. The original R^2^Y and Q^2^-values were higher than those obtained after 500 permutations, and the regression line of Q^2^-points intersected the vertical axis below zero (−0.126; Figure 3A). The RMSEE and the RMSECv were close (109.4 and 111.5, respectively, Figure 4A).

A group of 64 buckets with a VIP > 1 explained the first latent variable (Supplementary Data 1). For the buckets with VIP > 1 and BH *p* < 0.05, the involved metabolites were identified manually by importing chemical shift lists into the Human Metabolome Database (Wishart et al., 2009; See Footnote 3). They corresponded to 14 metabolites summarized in Table 1. There were seven carbohydrates identified as biomarkers for LW. For each metabolite, the number of peaks that contained at least one bucket with VIP > 1 was respectively, seven out of 10 peaks for glucose-6-phosphate (HMDB0001401), six out of eight peaks for glucuronic acid (HMDB0000127), all the two peaks for glyceric acid (HMDB0000139), all two peaks for glycogen (HMDB0000757), all the two peaks for lactate (HMDB0000190), two out of four peaks for malic acid (HMDB0000156), and 12 out of 13 peaks for maltose (HMDB0000163; Table 1). There were five amino acids identified as biomarkers of LW. For each metabolite, the number of peaks that contained at least one bucket with VIP > 1 was, respectively, two out of four ^1^H-NMR peaks for arginine (HMDB0000517), one out of two peaks for N-acetylglycine (HMDB0000532), three out of five peaks for proline (HMDB0000162), all the two peaks for taurine (HMDB0000251) and six out of seven peaks for trans-4-hydroxy-L-proline (HMDB0000725; Table 1). There were also two other organic compounds identified as biomarkers of LW. For each metabolite, the number of peaks that contained at least one bucket with VIP > 1 was the one peak for allantoin (HMDB0000462) and three out of four peaks for glycerophosphocholine (HMDB0000086; Table 1).

**Table 1:** List of the 14 biomarkers of *foie gras* liver weight identified with the bucket method.

Metabolites	^1^H-NMR peak^γ^	Chemical shift^δ^ (ppm)	number of buckets with VIP > 1^ε^	BH *p*-value^ζ^	BH *p*-value 2^η^
Carbohydrate					
**Glucose-6-phosphate**					**0.237**
HMDB0001401	multiplet	3.26–3.28	4	<0.001–0.01	
	multiplet	3.47–3.51	7	<0.001–0.004	
	multiplet	3.55–3.59	5	<0.001–0.002	
	triplet	3.70–3.73	4	<0.001	
	doublet	3.87–3.88	0		
	multiplet	3.90–3.94	2	<0.001	
	triplet	3.98–4.00	0		
	multiplet	4.02–4.05	2	<0.001	
	doublet	4.63–4.64	0		
	singlet	5.22	2	<0.001	
**Glucuronic acid**					**<0.001**
HMDB0000127	multiplet	3.27–3.30	4	<0.001–0.010	
	multiplet	3.49–3.54	7	<0.001–0.004	
	quartet	3.57–3.59	3	<0.001–0.006	
	multiplet	3.72–3.75	8	<0.001–0.040	
	doublet	4.08–4.09	2	<0.001–0.005	
	singlet	4.64	0		
	singlet	4.66	0		
	doublet	5.24–5.25	2	<0.001	
**Glyceric acid**					**<0.001**
HMDB0000139	multiplet	3.72–3.84	12	<0.001–0.040	
	multiplet	4.12–4.14	2	<0.001	
**Glycogen**					**0.070**
HMDB0000757	singlet	3.83	3	<0.001	
	singlet	5.39	1	0.030	
**Lactate**					**<0.001**
HMDB0000190	doublet	1.31–1.32	3	<0.001	
	quartet	4.08–4.12	5	<0.001–0.005	
**Malic acid**					**<0.001**
HMDB0000156	quartet	2.33–2.38	4	<0.001–0.050	
	doublet	2.64–2.65	0		
	doublet	2.67–2.68	0		
	quartet	4.28–4.31	1	<0.001	
**Maltose**					**<0.001**
HMDB0000163	singlet	5.40	2	<0.001–0.030	
	doublet	5.22–5.23	2	<0.001	
	doublet	3.96–3.98	1	<0.001	
	doublet	3.93–3.94	1	<0.001	
	doublet	3.89–3.92			
	multiplet	3.81–3.87	5	<0.001	
	multiplet	3.74–3.79	6	<0.001–0.040	
	multiplet	3.69–3.73	4	<0.001	
	quartet	3.65–3.68	1	0.005	
	singlet	3.63	1	0.005	
	multiplet	3.60–3.55	5	<0.001–0.002	
	triplet	3.43–3.39	5	<0.001	
	quartet	3.25–3.28	5	<0.001–0.200	
Amino acids					
**Arginine**					**0.004**
HMDB0000517	muitiplet	1.605–1.756	1		
	muitiplet	1.874–1.935	0		
	triplet	3.248–3.220	0		
	triplet	3.769–3.744	5	<0.001	
**N-acetylglycine**					**0.141**
HMDB0000532	singlet	8	0		
	doublet	3.745–3.768	6	<0.001–0.040	
**Proline**					**0.237**
HMDB0000162	multiplet	1.94–2.09	0		
	multiplet	2.31–2.37	4	<0.001–0.050	
	multiplet	3.30–3.35	2	<0.002	
	multiplet	3.38–3.42	0		
	multiplet	4.11–4.13	1	<0.001	
**Taurine**					**<0.001**
HMDB0000251	triplet	3.24–3.26	6	<0.001–0.010	
	triplet	3.40–3.43	6	<0.001	
**trans-4-hydroxy-L-proline**					**0.005**
HMDB0000725	quartet	4.320–4.350	0		
	doublet	3.480–3.490	4	<0.001–0.001	
	singlet	3.46	1	<0.001	
	singlet	3.37	1	<0.001	
	doublet	3.340–3.350	1	<0.001	
	multiplet	2.390–2.450	2	<0.001–0.050	
	multiplet	2.120–2.170	1	<0.001	
Organic compounds					
**Allantoin**					**0.020**
HMDB0000462	singlet	5.38	1	0.030	
**Glycerophosphocholine**					**0.070**
HMDB0000086	singlet	3.20	7	<0.001–0.200	
	multiplet	3.59–3.68	4	<0.001–0.006	
	multiplet	3.84–3.95	8	<0.001	
	quartet	4.29–4.33	0		

γ For each metabolite, the nature of each ^1^H-NMR peak is mentioned.

δ For each metabolite, the range of chemical shift of each peak is mentioned in ppm.

ε The PLS model to describe the liver weight with bucket data was plotted. The first latent variable enabled to separate the fatty livers in function of their liver weight. The VIP of the buckets involved in the first latent were extracted. For each ^1^H-NMR peak of each metabolite, the number of buckets with VIP > 1 was indicated.

ζ For each bucket, the effect of the bucket value on the liver weight was tested by a linear model with R software, and the *p*-values were corrected with the Benjamini-Hochberg procedure and named BH *p*-values. For each metabolite, the range of BH *p*-values of each peak was mentioned.

η For each biomarker, the relative metabolite concentration was computed with the bucket data. A linear model with R software tested the effect of the relative metabolite concentration on the liver weight, and the *p*-values were corrected with the Benjamini-Hochberg procedure and named BH *p*-values 2.

The relative concentrations (RC) of all the 14 metabolites were computed. The BH *p*-values were summarized in Table 1. A total of nine metabolites were statistically significant (BH *p* < 0.05) and two tended to be significant (*p* < 0.10). All these 11 metabolites were further considered as LW biomarkers identified by the bucket method. There were six out the seven carbohydrates: glucuronic acid (*p* < 0.001), glyceric acid (*p* < 0.001), glycogen (*p* = 0.070), lactate (*p* < 0.001), malic acid (*p* < 0.001) and maltose (*p* < 0.001) for carbohydrates, three out of five amino acids: arginine (*p* < 0.001), taurine (*p* < 0.001), and trans-4-hydroxy-L-proline (*p* = 0.005) and also allantoin (*p* = 0.020) and glycerophosphocholine (*p* = 0.070; Table 1).

In parallel, the metabolite method was applied, and the 64 spectra were converted into a table of 41 metabolite values with the ASICS R package. No outlier was detected by PCA. The PLS scatter plot had only one latent variable, and the parameters of the models were cumulative R^2^X = 0.534 and R^2^Y = 0.650 (Figure 2B). The prediction of the model was low Q^2^ = 0.626. The evolution of LW was well represented on the vertical axis corresponding to the first latent variable (Figure 2B). The original R^2^Y and Q^2^-values were higher than those obtained after 500 permutations, and the regression line of Q^2^-points intersected the vertical axis below zero (−0.119; Figure 3B). The RMSEE and the RMSECv were close (110.6 and 112.5, respectively, Figure 4B). The evolution of LW was well represented on the vertical axis corresponding to the first latent variable (Figure 2B). Only five metabolites had a VIP > 1 to explain this axis, of which all had a BH *p* < 0.05 (Table 2). Including lactate (VIP = 4.11, BH *p* < 0.001), glucose (VIP = 2.87, BH *p* < 0.001), threonine (VIP = 1.72, BH *p* < 0.001), alanine (VIP = 1.55, BH *p* < 0.001) and taurine (VIP = 1.29, BH *p* < 0.001; Table 2).

**Table 2:** List of the five biomarkers of *foie gras* liver weight identified with the metabolite method.

Metabolites	VIP-values^ε^	BH *p*-values^ζ^	R^2^
Lactate	4.11	<0.001	0.65
Glucose	2.87	<0.001	0.51
Threonine	1.72	<0.001	0.62
Alanine	1.55	<0.001	0.35
Taurine	1.29	<0.001	0.26

ε The PLS model to describe the liver weight with metabolite data was plotted. The first latent variable enabled to separate the fatty livers in function of the liver weight. The metabolites with VIP superior to 1 were selected. The VIP of the metabolite was indicated.

ζ For each biomarker, the effect of their relative concentration on the liver weight was tested by a linear model with R software, and *p*-values were corrected with the Benjamini-Hochberg procedure and named BH *p*-values.

**Figure 5:**
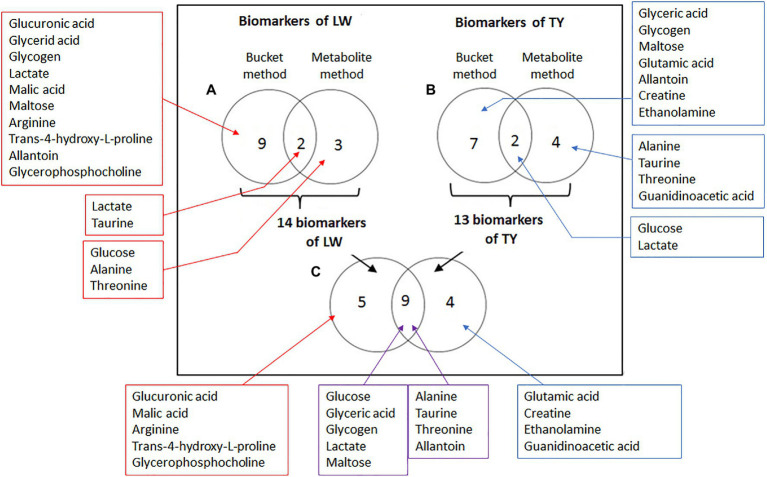
Comparisons of biomarker lists with Venn diagram. **(A)** Biomarkers of liver weight identified by the bucket method and by the metabolite method (with VIP > 1 and BH *p*-value < 0.1). **(B)** Biomarkers of technological yield identified by the bucket method and by the metabolite method. **(C)** Biomarkers of liver weight and technological yield identified by at least one method.

**Figure 6:**
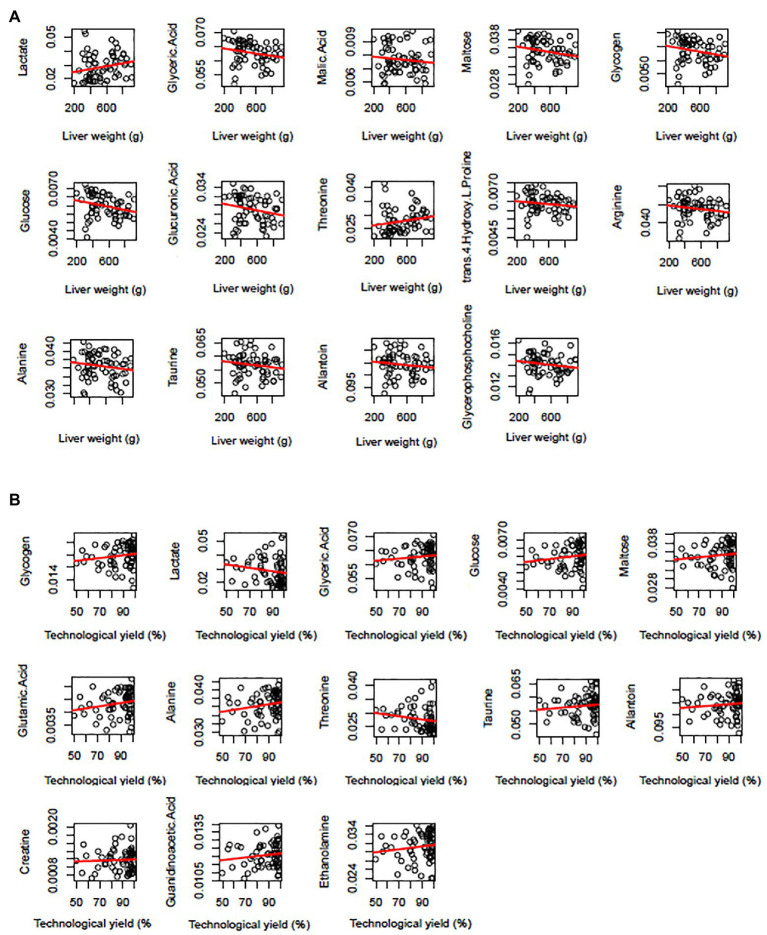
Plots of biomarker relative contents in function of liver weight **(A)** or liver technological yield **(B)**. The metabolite relative contents were computed with the bucket data and had no unit. The regression curves were in red.

**Figure 7:**
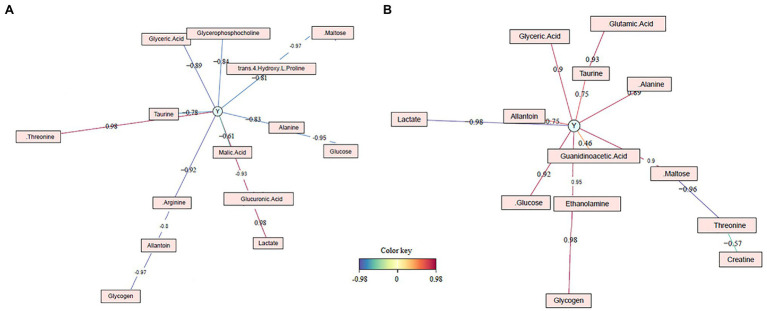
Correlation networks of *foie gras* biomarkers **(A)** of liver weight (Y represents the liver weight) and **(B)** of liver technological yield (Y represents the technological yield). The relative concentration used for the correlations are calculated with the bucket data.

In conclusion, for LW, there were 14 biomarkers. Two biomarkers were identified by the bucket method and the metabolite method (lactate, taurine), three were identified only by the metabolite method (glucose, alanine, and threonine), and nine metabolites were only identified by the bucket method (glucuronic acid, glyceric acid, glycogen, malic acid, maltose, arginine, trans-4-hydroxy-L-proline, allantoin, and glycerophosphocholine; Figure 5A). For the 14 biomarkers, their RCs were computed with the bucket data, and the plots of their RCs in the function of LW were presented in Figure 6A. The correlation network between LW and the biomarkers was presented in Figure 7A. LW was strongly negatively correlated with glucose (−0.95), glycogen (−0.97), glucuronic acid (−0.93), glyceric acid (−0.89), malic acid (−0.61), and maltose (−0.97) and also to alanine (−0.83), arginine (−0.92), taurine (−0.78), trans-4-hydroxy-L-proline (−0.81), and to allantoin (−0.80) and glycerophosphocholine (−0.84). On the contrary, TY was strongly correlated with lactate (0.98) and threonine (0.98; Figure 7A).

### Identification of Hepatic Biomarkers of *Foie Gras* Technological Yield

First, the spectra were analyzed with the bucket method. A PCA was first performed, but no outlier was detected (not shown). The PLS scatter plot to explain TY had only one latent variable, and the parameters were cumulative R^2^X = 0.506 and R^2^Y = 0.514. The prediction of the model was Q^2^ = 0.471. The original R^2^Y and Q^2^-values were higher than those obtained after 500 permutations, and the regression line of Q^2^-points intersected the vertical axis below zero (−0.114; Figure 3C). The RMSEE and the RMSECv were close (8.9 and 9.2, respectively; Figure 4C). The projection of the samples highlighted an evolution of TY with the first latent variable on the vertical axis (Figure 2C). A total of 64 buckets had a VIP value superior to 1 (Supplementary Data 2). They corresponded to 14 metabolites. All the buckets corresponding to each 1H-NMR peak were identified for each metabolite, and their VIP values and their BH *p*-values were summarized in Table 3. There were six carbohydrates identified as biomarkers for TY. For each metabolite, the number of peaks that contained at least one bucket with VIP > 1 was, respectively, 21 out of 22 peaks for glucose (HMDB0000122), eight out of 10 peaks for glucose-6-phosphate (HMDB0001401), all of the two peaks for glyceric acid (HMDB0000139), all the two peaks for glycogen (HMDB0000757), all the two peaks for lactate (HMDB0000190) and 12 out of 13 peaks for maltose (HMDB0000163). There were also five amino acids identified as biomarkers of TY of *foie gras*. For each metabolite, the number of peaks that contained at least one bucket with VIP > 1 was, respectively, three out four peaks for arginine (HMDB0000517), eight out of nine peaks for glutamic acid (HMDB0000148), one out of two peaks for N-acetylglycine (HMDB0000532), all the five peaks for proline (HMDB0000162) and all the seven peaks for trans-4-hydroxy-L-proline (HMDB0000725). There were also three other organic compounds identified as biomarkers of TY. For each metabolite, the number of peaks that contained at least one bucket with VIP > 1 was, respectively, the only one peak for allantoin (HMDB0000462), all the two peaks for creatine (HMDB0000064), and all the two peaks for ethanolamine (HMDB0000149; Table 3).

**Table 3:** List of the 14 biomarkers of *foie gras* technological yield identified with the bucket method.

Metabolites	^1^H-NMR peak^γ^	Chemical shift^δ^ (ppm)	number of buckets with VIP > 1^ε^	BH *p*-value^ζ^	BH *p*-value 2^η^
Carbohydrate					
**Glucose**					**<0.001**
HMDB0000122	quartet	3.22.25	4	<0.001 to 0.005	
	singlet	3.38	1	<0.001	
	doublet	3.39	2	<0.001	
	doublet	3.40–3.41	1	<0.001	
	singlet	3.42	1	<0.001	
	doublet	3.43–3.44	2	<0.001	
	quartet	3.45–3.46	3	<0.001	
	quartet	3.46	1	<0.001	
	singlet	3.48	1	<0.001	
	singlet	3.49	3	<0.001 to 0.030	
	doublet	3.51–3.52	3	<0.001 to 0.050	
	doublet	3.53–3.54	4	<0.001 to 0.050	
	quartet	3.69–3.71	4	<0.001 to 0.030	
	multiplet	3.72–3.77	7	<0.001	
	doublet	3.80–3.81	2	<0.001	
	singlet	3.82	2	<0.001	
	doublet	3.82–3.83	3	<0.001	
	doublet	3.84–3.85	4	<0.001	
	doublet	3.87–3.88	2	<0.001	
	doublet	3.89–3.90	2	<0.001	
	doublet	4.63–4.64	0		
	doublet	5.22	2	<0.001	
**Glucose-6-phosphate**					**0.490**
HMDB0001401	multiplet	3.26–3.28	4	<0.001 to 0.080	
	multiplet	3.47–3.51	7	<0.001 to 0.030	
	multiplet	3.55–3.59	4	0.005 to 0.040	
	triplet	3.70–3.73	4	<0.001	
	doublet	3.87–3.88	2	<0.001	
	multiplet	3.90–3.94	2	<0.001 to 0.020	
	triplet	3.98–4.00	3	<0.001 to 0.020	
	multiplet	4.02–4.05			
	doublet	4.63–4.64	0		
	singlet	5.22	2	<0.001	
**Glyceric acid**					**<0.001**
HMDB0000139	multiplet	3.72–3.84	11	<0.001	
	multiplet	4.12–4.14	2	<0.001 to 0.050	
**Glycogen**					**0.011**
HMDB0000757	singlet	3.83	4	<0.001	
	singlet	5.39	2	0.001 to 0.007	
**Lactate**					**<0.001**
HMDB0000190	doublet	1.31–1.32	2	<0.001	
	quartet	4.08–4.13	5	<0.001 to 0.050	
**Maltose**					**<0.001**
HMDB0000163	singlet	5.4	2	0.001 to 0.007	
	doublet	5.22–5.23	2	<0.001	
	doublet	3.96–3.98	2	<0.001 to 0.020	
	doublet	3.93–3.94			
	doublet	3.89–3.92	3	<0.001	
	multiplet	3.87–3.81	5	<0.001	
	multiplet	3.74–3.79	5	<0.001	
	multiplet	3.69–3.73	7	<0.001 to 0.030	
	quartet	3.65–3.68	4	<0.001 to 0.030	
	singlet	3.63	1	0.010	
	multiplet	3.60–3.55	4	0.005 to 0.040	
	triplet	3.39–3.43	5	<0.001	
	quartet	3.28–3.25	5	<0.001 to 0.080	
Amino acids					
**Arginine**					**0.490**
HMDB0000517	multiplet	1.60–1.75	1	0.002	
	multiplet	1.87–1.93	0		
	triplet	3.22–3.25	6	<0.001 to 0.080	
	triplet	3.74–3.77	5	<0.001	
**Glutamic acid**					**0.010**
HMDB0000148	quartet	3.73–3.76	5	<0.001	
	multiplet	2.00–2.15	1	0.006	
	singlet	2.29	1	0.005	
	singlet	2.31	1	0.005	
	doublet	2.32–2.33	1	0.005	
	doublet	2.34	1	0.005	
	doublet	2.35–2.36	2		
	singlet	2.38	2	<0.001 to 0.005	
	singlet	2.39	2	<0.001 to 0.110	
**N-acetylglycine**					**0.495**
HMDB0000532	singlet	8	0		
	doublet	3.75–3.77	5	<0.001	
**Proline**					**0.286**
HMDB0000162	multiplet	1.94–2.09	1	0.006	
	multiplet	2.31–2.37	3	<0.001 to 0.005	
	multiplet	3.30–3.35	2	<0.001	
	multiplet	3.38–3.42	5	<0.001	
	multiplet	4.11–4.13	3	<0.001 to 0.050	
**trans-4-hydroxy-L-proline**					**0.147**
HMDB0000725	multiplet	2.12–2.17	2	0.004 to 0.006	
	multiplet	2.39–2.45	3	<0.001 to 0.100	
	doublet	3.34–3.35	1	<0.001	
	singlet	3.37	1	<0.001	
	singlet	3.46	2	0.010	
	doublet	3.48–3.49	4	<0.001 to 0.030	
	quartet	4.32–4.35	1	0.001	
Organic compound					
**Allantoin**					**0.004**
HMDB0000462	singlet	5.38	2	0.001–0.007	
**Creatine**					**<0.001**
HMDB0000064	singlet	3.02	1	<0.001	
	singlet	3.92	3	<0.001–0.020	
**Ethanolamine**					**0.009**
HMDB0000149	triplet	3.12–3.14	1	<0.001	
	triplet	3.80–3.82	3	<0.001	

γ For each metabolite, the nature of each ^1^H-NMR peak is mentioned.

δ For each metabolite, the range of chemical shift of each peak is mentioned in ppm.

ε The PLS model to describe the technological yield with bucket data was plotted. The first latent variable enabled to separate the fatty livers in function of the technological yield. The VIP of the buckets involved in the first latent were extracted. For each 1H-NMR peak of each metabolite, the number of buckets with VIP > 1 was indicated.

ζ For each bucket, the effect of the bucket value on the technological yield was tested by a linear model with R software, and the *p-values* were corrected with the Benjamini–Hochberg procedure and named BH *p*-values. For each metabolite, the range of BH *p*-values of each peak was mentioned.

η For each biomarker, the relative metabolite concentration was computed with the bucket data. A linear model with R software tested the effect of the relative metabolite concentration on the technological yield, and the *p-values* were corrected with the Benjamini–Hochberg procedure and named BH p-values 2.

The RCs of all the 14 metabolites were computed. The BH *p*-values were summarized in Table 3. A total of nine metabolites were considered as statistically significant (BH *p* < 0.05) whose glucose, glyceric acid, glycogen, lactate, maltose for carbohydrates, only glutamic acid for the amino acids and allantoin, creatine, and ethanolamine (Table 2). All these nine metabolites were further considered as biomarkers of TY.

In parallel, the metabolite method was performed. The individuals were less well represented on the scatter plot than with the bucket data (1 latent variable, cumulative R^2^X = 0.533, R^2^Y = 0.522; Figure 2D). The prediction of the model was Q^2^ = 0.418. The original R^2^Y and Q^2^-values were higher than those obtained after permutation, and the regression line of Q^2^-points intersected the vertical axis below zero (−0.104; Figure 3D). The RMSEE and the RMSECv were close (8.9 and 9.1, respectively; Figure 4D). The latent variable on the vertical axis explained the evolution of TY (Figure 2D), and six metabolites had VIP values superior to 1 (Table 4). Including glucose (VIP = 2.82, BH *p* < 0.001), lactate (VIP = 4.06, BH *p* < 0.001), alanine (VIP = 1.43, BH *p* < 0.001), taurine (VIP = 1.51, BH *p* < 0.001), threonine (VIP = 1.72, BH *p* < 0.001) and guanidinoacetic acid (VIP = 1.04, BH *p* < 0.001).

**Table 4:** List of the 6 biomarkers of *foie gras* technological yield identified with the metabolite method.

Var ID (Primary)	VIP-values^ε^	BH *p*-values^ζ^	R^2^
Glucose	2.8	<0.001	0.39
Lactate	4.06	<0.001	0.50
Alanine	1.43	<0.001	0.24
Taurine	1.51	<0.001	0.28
Threonine	1.72	<0.001	0.49
Guanidinoacetic acid	1.04	<0.001	0.39

ε The PLS model to describe the technological yield with metabolite data was plotted. The first latent variable enabled to separate the fatty livers in function of the liver weight. The metabolites with VIP superior to 1 were selected. The VIP of the metabolite was indicated.

ζ For each biomarker, the effect of their relative concentration on the technological yield was tested by a linear model with R software, and the *p*-values were corrected with the Benjamini-Hochberg procedure and named BH *p*-values.

In conclusion, for TY, there were 13 biomarkers: two biomarkers were identified by the bucket method and the metabolite method (glucose and lactate), four were identified only by the metabolite method (alanine, taurine, threonine, and guanidino acetic acid), and seven metabolites were identified by the bucket method (glyceric acid, glycogen, maltose, glutamic acid, allantoin, creatine, and ethanolamine; Figure 5B).

For the 13 biomarkers of TY, their RC was computed with the bucket data, and the plots of their RC in the TY function were presented in Figure 6B. The correlation network between TY and the biomarker RC was presented in Figure 7B. TY was positively correlated with glucose (0.92), glycogen (0.98), glyceric acid (0.9), maltose (0.9), and also to alanine (0.89), glutamic acid (0.93), taurine (0.75), and to allantoin (0.75), ethanolamine (0.95), and guanidinoacetic acid (0.46), whereas TY was negatively correlated with lactate (−0.98), threonine (−0.96) and creatine (−0.57; Figure 7B).

Consequently, the results of the ^1^H-NMR analysis identified 14 hepatic biomarkers for the *foie gras* liver weight and 13 for its technological yield (Table 5 and Figures 5A,B). As the phenotypic correlation between LW and TY was strong (−0.80, *p* < 0.001), nine biomarkers were common to LW and TY (Figure 5C), of which five carbohydrates: glucose, glyceric acid, glycogen, lactate, maltose, three amino acids: alanine, taurine, threonine, and allantoin. There were five biomarkers specific to LW: glucuronic acid, malic acid, arginine, trans-4-hydroxy-L-proline, glycerophosphocholine, and four biomarkers specific to TY: glutamic acid, creatine, ethanolamine, and guanidinoacetic acid (Figure 5C).

**Table 5:** List of the biomarkers of liver weight and technological yield of *foie gras*.

	Biomarkers of liver weight						Biomarkers of technological yield					
	with bucket method			with metabolite method			with bucket method			with metabolite method		
	Important peaks^ε^	BH *p*-Value^ζ^	correlation with LW^η^	VIP^δ^	BH *p*-Value^ζ^	correlation with LW^η^	Important peaks^ε^	BH *p*-Value^ζ^	correlation with TY^η^	VIP^δ^	BH *p*-Value^ζ^	correlation with TY^η^
Biomarkers of LW and TY												
Alanine			−0.83	1.55		−0.9			0.89	1.43	<0.001	0.81
Allantoin	1/1	0.020	−0.8				1/1	0.004	0.75			
Glucose				2.87	<0.001	−0.95	21/22	<0.001	0.92	2.86	<0.001	0.94
Glyceric acid	2/2	<0.001	−0.89				2/2	<0.001	0.90			
Glycogen	2/2	0.007	−0.97				2/2	0.01	0.98			
Lactate	2/2	<0.001	0.98	4.11	<0.001	0.94	2/2	<0.001	−0.98	4.06	<0.001	−0.97
Maltose	12/12	<0.001	−0.97				12/12	<0.001	0.90			
Taurine	2/2	<0.001	−0.78	1.29	<0.001	−0.84			0.75	1.51	<0.001	0.82
Threonine			0.98	1.72	<0.001	0.96			−0.96	1.72	<0.001	−0.95
Biomarkers of LW												
Arginine	2/3	0.004	−0.92									
Glucuronic acid	6/8	<0.001	−0.93									
Glycerophosphocholine	3/4	0.070	−0.84									
Malic acid	2/4	<0.001	−0.61									
Trans-4-hydroxy-L-proline	6/7	0.005	−0.81									
Biomarkers of TY												
Creatine							2/2	<0.001	−0.57			
Ethanolamine							2/2	0.009	0.95			
Glutamic acid							8/8	0.01	0.93			
Guanidinoacetic acid									0.46	1.04	<0.001	0.86

ε For each biomarker, the number of important peaks compared with the total number of 1H-NMR peaks is indicated. The important peaks contained at least one bucket with a VIP > 1 to explain the first latent variable of the PLS model of liver weight or technological yield.

ζ The models of the effects of the relative metabolite concentration on the liver weight and technological yield were computed. The *p*-values were corrected with the Benjamini–Hochberg procedure and indicated.

η The Pearson correlation of the metabolite relative concentration obtained with bucket data or metabolite data and the liver weight or the technological yield was indicated.

δ The PLS model to describe the liver weight or the technological yield with metabolite data was plotted. The first latent variable enabled to separate the fatty livers in function of the liver weight or the technological yield. The metabolites with VIP superior to 1 were selected. The VIP of the metabolite was indicated.

## Discussion

In this experimental design, the ducks were overfed for 6 to 12 days and received 11 to 23 meals. As a result, large variations in duck body weights, LW, and liver TY occurred, explaining the high correlations observed between the biomarker relative quantities and the liver characteristics.

During the overfeeding, the feed was based on corn. As corn is a cereal, it is rich in starch (around 63% as fed). The ingested starch is converted into glucose in the small intestine. Then the glucose is absorbed into the hepatic portal vein and carry to the liver. There, it can be stored like glycogen or converted into fatty acids by *de novo* lipogenesis. In the study, the liver weight was highly increased (from 80 g on day 0 to more than 750 g on day 12). Thus, the plasmatic glucose contents measured in the carotid artery corresponded to the result of the glucose transfer into the portal vein and the glucose uptake by the liver and other organs. However, the plasmatic glucose content decreased during the overfeeding while LW was increased (correlation of −0.94 in Mozduri et al., 2021) although the ingested starch was strongly increased as the corn quantity meal was increased. That means that the plasmatic glucose content was up-taken more efficiently by the liver during the overfeeding (Pioche et al., 2019). On the contrary, the glucose content in the liver was decreased when LW was increased with a negative correlation of −0.95 between them. So, the glucose was highly metabolized in the liver. At the beginning of the overfeeding period, the liver metabolism was more oriented through glycogenogenesis with strong glycogen storage (until 106 mg of glycogen by grams of the liver after three meals at day 2). In contrast, after seven meals on day 4, the liver metabolism shifted to lipogenesis with a strong triglyceride accumulation (29.5% of lipids at day 4 vs. 4.6 at day 0; Bonnefont et al., 2019). The glycogen content estimated in the liver in the second half of the overfeeding (from day 6 to day 12) was strongly negatively correlated with liver weight (−0.97). Furthermore, in other animal models as rats with hepatic steatosis, the liver glycogen content was lower than in control rats (Kusunoki et al., 2002).

In addition, LW was negatively correlated with TY (−0.82). Thus, the glucose content and the glycogen content were strongly correlated with TY (+0.92 and +0.98). That is consistent with previous results on *foie gras* with livers weighing around 580 g (Bonnefont et al., 2014).

In parallel, the hepatic lactate content was the most important metabolite to discriminate the livers in the function of their LW or TY (VIP values of the O-PLS models of 4.11 and 4.06, respectively). It was positively correlated with LW (+0.98) and negatively correlated with TY (−0.98). Previously, the lactate was the most discriminant metabolite between high TY livers and low TY livers with equivalent weights (Bonnefont et al., 2014). The lactate is the last metabolite of anaerobic glycolysis that converts glucose into pyruvate and then into lactate *via* the lactate dehydrogenase enzyme. In a recent mouse model, it was shown that glucose oxidation in the liver was central in the development of steatosis, as glycolysis metabolizes glucose into pyruvate, which can be anaerobically converted into lactate or aerobically converted into acetyl-CoA. The hepatic lactate in nonalcoholic steatohepatitis (NASH) was primarily diverted toward the production of acetyl-CoA for lipogenesis rather than the production of glucose (Zhu et al., 2018). In addition, Lo et al. (2020b) recently demonstrated in ducks undergoing overfeeding that low LW was associated with aerobic energy metabolism and high weight livers with anaerobic energy metabolism (Lo et al., 2020b). These results suggest that the efficiency of energy metabolism would influence both LW and TY. Therefore, it can be supposed that the increase in LW could lead to a decrease in the efficiency of this metabolism translated by the increase in hepatic lactate content, which would result in a decrease in TY.

Contrary to hepatic lactate content, the glucuronic acid hepatic content was negatively correlated with LW (−0.93). However, the glucuronic acid can be conjugated to lipophilic substrates *via* UDP-glucuronosyltransferases. These enzymes catalyze phase II biotransformation reactions and promote glucuronidation. Glucuronidation is a major detoxification pathway for endogenous and exogenous compounds, and it is involved in transporter-mediated excretion into bile and urine (Williams et al., 2004). In steatosis livers of mice, the UDP-glucuronosyltransferase expression increased with the increased hepatic triglyceride content and could have a significant impact on determining circulating hormone levels (Xu et al., 2012). Thus, the lower quantity of glucuronic acid in the high-weight liver could be explained using glucuronic acid for glucuronidation.

The amino acid metabolism of duck livers was strongly impacted by the overfeeding period at the same time as the carbohydrate metabolism. The alanine, taurine, and threonine were identified as biomarkers of both LW and TY, whereas arginine and trans-4-hydroxy-proline were only identified as biomarkers of LW and glutamic acid of TY. Briefly, the amino acid liver content was decreased when the LW was increased and/or when the TY was decreased except for the threonine. The reduction of amino acid metabolism in the liver with the enhanced hepatic steatosis was already underlined in overfed ducks (François et al., 2014; Lo et al., 2020b) and other hepatic steatosis models as obese mice (Du et al., 2012; Bruckbauer and Zemel, 2014). Thus, these amino acids were increased in low-weight livers. It was shown in rats and mice that a diet supplementation in taurine (Gentile et al., 2011) and arginine (Voloshin et al., 2014; Sellmann et al., 2017) reduced hepatic lipid accumulation. Thus, they were used as a preventative treatment against NASH. The taurine transporter-deficient mice showed strongly decreased taurine levels in various tissues like the liver and developed chronic hepatitis and liver fibrosis during adulthood, accompanied by severe augmentation of hepatocyte apoptosis (Warskulat et al., 2006). In addition, the restriction of lysine and threonine in diets increased the free fatty acid content in the liver of rats (Viviani et al., 1966).

Furthermore, the increase of the activities of the transaminase enzymes alanine aminotransferase (ALT) and aspartate aminotransferase (AST) with liver injury is strongly documented (Pratt and Kaplan, 2000). ALT catalyzes the transfer of an amino group from alanine to α-ketoglutarate to produce pyruvate and glutamate, and AST catalyzes the interconversion of aspartate and α-ketoglutarate to oxaloacetate and glutamate. Both reactions are reversible. Thus, the identification of alanine as a biomarker of both LW and TY and glutamic acid as a specific biomarker of TY should be directly linked to the ALT and AST activities in the liver. Also, the oxaloacetate issued from AST activity is a key intermediate in the citric acid cycle. Actually, in this cycle, the malate dehydrogenase converts the malate into oxaloacetate with the NAD^+^ cofactor, and after several reactions, the oxaloacetate is converted again into malate. However, the malate is known to play a role in lipogenesis by furnishing NADPH to reduce acetyl CoA to fatty acids (Wise Jr and Ball, 1964). This may explain the identification of malic acid as a biomarker of LW. In the Poland goose, the activity of ME was correlated positively with the weight of the fatty liver (Mourot et al., 2000).

In addition, glyceric acid was identified as a biomarker of LW and TY. The glyceric acid is a substrate for glycerol synthesis (Feraudi and Bert, 1977), and the glycerol is a substrate for triglyceride synthesis (Tidwell and Johnston, 1961). Thus, the negative correlation of glyceric acid with LW (−0.89) could be explained by using glyceric acid for lipogenesis.

Moreover, in the liver, creatine is synthesized from glycine and arginine. First, both amino acids are combined to form guanidinoacetate which is then methylated using S-adenosyl methionine to synthesize creatine (Rosenberg, 1959). The amount of arginine was negatively correlated with LW (−0.92). The arginine is involved in the process of creatine synthesis (Barcelos et al., 2016). Creatine plays a role in the antioxidant role against aqueous radical and reactive species ions (Lawler et al., 2002). The supplementation in creatine protects the liver from hepatotoxicity by attenuating oxidative stress (Aljobaily et al., 2021). Lo et al. (2020a) showed that, in overfed liver duck, an increase in liver weight resulted in a rise in the cellular oxidative stress level (Lo et al., 2020a). These results corroborated the positive correlation between the level of oxidative stress and LW.

The creatine phosphate serves as a dynamic reservoir of high-energy phosphate in exchange for ATP. The steatosis strongly impacts creatine metabolism as arginine is a specific biomarker of LW, and guanidinoacetate and creatine are specific biomarkers of TY. However, contrary to guanidinoacetate, creatine was negatively correlated with TY. Thus, the creatine content in the liver with low TY may be increased to control oxidative stress. Oxidative stress is defined as the presence of metabolic and radical substances or so-called reactive (oxygen, nitrogen, or chlorine) species (Elnesr et al., 2019; Elwan et al., 2019).

Furthermore, the positive correlation of hepatic amino acid content and TY in *foie gras* was previously underlined in association with a reduction of oxidative stress (Theron et al., 2011; Bonnefont et al., 2014; François et al., 2014). Actually in chronic liver disease there is increased reactive oxygen species production and decreased activity of antioxidant systems (De Minicis et al., 2006). Recently, hypoxic response to severe hypoxia was highlighted in heavy livers of ducks (Lo et al., 2020a).

Moreover, the modification of amino acid metabolism in livers during the overfeeding period corroborates the evolution of amino acid metabolism in plasma of the same ducks (Mozduri et al., 2021) and other hepatic steatosis models, as it was discussed in Mozduri et al. (2021).

Finally, the glycerophosphocholine was identified as a specific biomarker of LW (−0.84), and ethanolamine as a specific biomarker of TY (+0.95). Ethanolamine can be phosphorylated to form phosphoethanolamine. Glycerophosphocholine and phosphoethanolamine are cytosolic intermediates of phospholipids synthesis. In patients with cirrhosis that is considered the most severe stage of liver steatosis disease, the phosphoethanolamine liver content was increased whereas the glycerophosphocholine was decreased compared to controls but not in patients with a lower severe stage of fatty liver disease as NASH (Sevastianova et al., 2010). On the contrary, Kalhan et al. (2011) reported a significantly lower glycerophosphocholine content in steatohepatitis compared with nondiabetic healthy controls in humans (Kalhan et al., 2011). Furthermore, in overfed ducks, a higher glycerophosphocholine content in the low-fat-loss livers that corresponded to high TY livers was underlined (Bonnefont et al., 2014).

The last biomarker of LW and TY was allantoin. It was shown that a supplementation in allantoin in NASH or diabetic mice inhibited the structural damage of the liver concerning fat accumulation (Movahhed et al., 2019; Ma et al., 2020) which explained its lower content in high weight livers.

## Conclusion

To conclude, the analysis of the metabolism of male mule duck livers during the overfeeding period through ^1^H-NMR analysis enabled the identification of eighteen liver biomarkers of *foie gras* qualities. Nine of them were identified for both LW and TY of *foie gras*: five were carbohydrates like glucose, glyceric acid, glycogen, lactate, and maltose, three were amino acids like alanine taurine and threonine, plus allantoin. Five of them were specific to the LW: two carbohydrates as glucuronic acid, malic acid, two amino acids as arginine, and trans-4-hydroxy-L-proline plus a glycerophosphocholine, a phospholipid. Furthermore, four biomarkers were specific to *foie gras* TY. Two of them were involved in creatine metabolism (creatine and guanidinoacetic acid). One could be an intermediate of phospholipid (ethanolamine) and may be involved in membrane stability. The last one was glutamic acid produced by ALT, whose activity in the liver is enhanced in hepatosteatosis. As a result, in heavy livers, the liver metabolism was oriented through a reduction of carbohydrate metabolism, and the plasma membrane could be damaged, which may explain the low technological yield of these livers.

These findings will be complete if an analysis of the correlation between the plasma and liver metabolisms is made to understand the co-evolution of these tissues during the overfeeding period.

## Data Availability Statement

The raw data supporting the conclusions of this article will be made available by the authors, without undue reservation.

## Ethics Statement

The animal study was reviewed and approved by experimental approval A24-137-1.

## Author Contributions

JA supervised the animal experimental rearing, overfeeding, and slaughtering. BL prepared the liver samples for NMR analysis with the supervision of NM-G. BL, NM-G, and CC performed the NMR analysis. BL and NM-G made the preprocessing of the NMR spectra. ZM, BL, and CB performed the statistical analyses to interpret the data that were helped by AB, MM, and AM, and wrote the paper. All authors contributed to the article and approved the submitted version.

### Conflict of Interest

The authors declare that the research was conducted in the absence of any commercial or financial relationships that could be construed as a potential conflict of interest.

## Footnotes

^1^https://workflow4metabolomics.org/

^2^https://bioconductor.org/packages/ASICS/

^3^http://hmdb.ca/

^4^https://bioinfogp.cnb.csic.es/tools/venny/index.html

^5^https://CRAN.R-project.org/package=mixOmics
